# Relationship between race and community water and sewer service in North Carolina, USA

**DOI:** 10.1371/journal.pone.0193225

**Published:** 2018-03-21

**Authors:** Hannah Gordon Leker, Jacqueline MacDonald Gibson

**Affiliations:** Department of Environmental Sciences and Engineering, Gillings School of Global Public Health, University of North Carolina, Chapel Hill, North Carolina, United States of America; University of Miami, UNITED STATES

## Abstract

Previous evidence has identified potential racial disparities in access to community water and sewer service in peri-urban areas adjacent to North Carolina municipalities. We performed the first quantitative, multi-county analysis of these disparities. Using publicly available data, we identified areas bordering municipalities and lacking community water and/or sewer service in 75 North Carolina counties. Logistic regression was performed to evaluate the relationship between race and access to service in peri-urban areas, controlling for population density, median home value, urban status, and percent white in the adjacent municipality. In the peri-urban areas analyzed, 67% of the population lacked community sewer service, and 33% lacked community water service. In areas other than those with no black residents, odds of having community water service (*p*<0.01) or at least one of the two services (*p*<0.05) were highest for census blocks with a small proportion of black residents and lowest in 100% black census blocks, though this trend did not hold for access to community sewer service alone. For example, odds of community water service were 85% higher in areas that were greater than 0% but less than 22% black than in 100% black areas (*p*<0.001). Peri-urban census blocks without black populations had the lowest odds of community water service, community sewer service, and at least one of the two services, but this difference was only statistically significant for sewer. Peri-urban areas lacking service with no black residents were wealthier than 100% black areas and areas with any percent black greater than 0%. Findings suggest two unserved groups of differing racial and socioeconomic status: (1) lower-income black populations potentially excluded from municipal services during the era of legal racial segregation and (2) higher-income non-black populations. Findings also suggest greater racial disparities in community water than community sewer services statewide.

## Introduction

The introduction of water and sewer services to the United States in the early 20^th^ century drastically reduced the incidence of waterborne disease. Clean water infrastructure, including water filtration and chlorination, contributed to approximately 50% of total observed mortality reduction and 75% of infant mortality reduction in large United States cities between 1900 and 1936 [1]. While this public health infrastructure has been instrumental in reducing waterborne disease incidence and mortality throughout the United States, there is evidence that peri-urban black communities located on the outskirts of North Carolina municipalities have been historically excluded from regulated community water and sewer services [2–6]. These communities rely instead on individual wells and septic systems despite their close proximity to municipal utilities. U.S. Environmental Protection Agency drinking water regulations do not apply to individual water sources such as wells. Even though installation is typically regulated by the state, well owners are responsible for their own well maintenance, water treatment, and monitoring [7].

Multiple previous studies suggest that individual on-site septic tanks and wells can pose higher health risks than treated and regulated community water and sewer services. Annual reports of United States drinking water outbreaks from 1971 to 2006 show that the proportion of outbreaks in public, shared water systems decreased over this 35-year time span, while the proportion in individual water systems increased [7]. A recent United States Geological Survey study of about 2,100 private wells in 48 states found that 23% had at least one chemical contaminant above health-based guidelines, 34% tested positive for total coliforms, and 8% were positive for *E*. *coli* [8]. In a study of a majority black, peri-urban North Carolina community lacking municipal services on the outskirts of Chapel Hill, 10 out 11 water wells tested exceeded at least one water quality standard, and only 47% of the 45 septic systems tested complied with all operation and maintenance guidelines [9]. A study of households in majority black, peri-urban communities of Wake County, North Carolina, relying on private wells found that 29.2% of 171 private well tap water samples tested positive for total coliform bacteria and 6.43% for *Escherichia coli*, compared with 0.556% and 0.00850% of municipal system samples [10].

Evidence of increased risks of drinking water contamination in private well water suggests that exclusion from nearby municipal water and sewer services could contribute to health disparities. However, the extent of racial disparities in access to community water and sewer service in peri-urban areas has not been systematically or quantitatively examined for the state of North Carolina, or any other state, as a whole. Previous research has shown that unincorporated communities at the fringes of cities and towns can have disparate characteristics; some are high-poverty areas, while others contain planned communities with new homes [11]. Additionally, the migration of white residents from urban centers to the suburbs over time has been documented [12]. The differences in municipal water and sewer services between predominantly black peri-urban communities and peri-urban communities with smaller black populations have not been studied. This research seeks to fill these knowledge gaps through a quantitative state-wide analysis of the relationship between race and access to community water and sewer service in peri-urban communities adjacent to municipalities. This research also responds to an in-person request for help from the former North Carolina Public Health Director, who prioritized redressing racial disparities in access to community water and sewer services in peri-urban areas and wanted to know the extent of these disparities. The objective of this study is to assess the relationship between race and access to community water and sewer services in peri-urban areas. In order to achieve this objective, publicly available databases reporting access to community water and sewer service in North Carolina were identified, and these databases were used to identify peri-urban communities lacking water and sewer services.

### Background: Municipal underbounding

In North Carolina, many people served by private wells and septic systems that may pose greater waterborne health risks live in rural areas where homes are far apart and extending water and sewer lines would be cost prohibitive. However, some communities relying on individual on-site water and sewer systems are in more densely populated regions just outside of municipal boundaries [2, 13]. Previous studies have documented racial minority groups that were excluded from municipal boundaries as the municipalities expanded around them. Geographer Charles Aiken proposed the term “underbounding” to describe this phenomenon [14]. In the southern United States these underbounded communities are mostly black [14–15].

Underbounded communities in North Carolina are often located in the extraterritorial jurisdiction (ETJ) of municipalities. The ETJ is an area outside of municipal limits that is still subject to a municipality’s development and planning regulations. ETJs extend one to three miles from municipal limits depending on municipality size [16]. Despite their close proximity to municipal boundaries and the municipal zoning power over these areas, municipal governments are not required to provide city services (including water and sewer service) to households in the ETJ, although they may choose to do so. In this research, the ETJ is used to represent peri-urban areas adjacent to North Carolina municipalities containing communities that may be underbounded and lacking community water and sewer services.

Prior case studies in North Carolina have examined the effects of municipal underbounding in the state [2, 13, 17, 18]. Thus far, most studies of racial disparities in access to community water and sewer service levels in North Carolina have been case studies in specific neighborhoods, cities, and counties. The case studies describe reliance on individual wells and septic systems that are often old and failing [3, 4, 6, 19]. A statistical analysis of the role of race in predicting community water service in Wake County, North Carolina, found that at the census block level, a 10% increase in proportion of a population classified as black increases the odds of not having community water service by 3.8% [5]. An analysis of the factors affecting access to community water and sewer services in three unincorporated communities in North Carolina found the primary barrier to extension of services to be access to funds to pay for extension of municipal water and sewer lines. In contrast, the health benefits of extending services were neither emphasized nor prioritized by most local decision makers, and failing systems were often under-reported [20].

Documented issues of social disparities in access to community water and sewer services also exist elsewhere in the United States. For example, some colonias in South Texas rely on water vending machines for drinking water due to a lack of community water services, and underbounding of some residents of colonias precludes them from political involvement in water supply decisions, limiting their access to domestic water supplies [21, 22]. Additionally, research in Texas’ Lower Rio Grande Valley has found that census blocks containing colonias are less likely to be annexed by municipalities than census blocks without colonias, which in turn limits their access to infrastructure such as community water and sewer services [23].

In addition to water and sewer access, social disparities in exposure to drinking water contaminants, a closely linked health concern, affect other regions. These concerns include water contamination in colonias along the US/Mexico border; unregulated water in the Navajo Nation; higher prevalence and duration of drinking water and boil water advisories for First Nations communities in Canada; Latino migrant worker communities in California’s Central Valley; and black residents in an unincorporated town in Texas, who are concerned about water quality and exposure to sewage [11, 24, 25].

While the project described in this article focuses on historical racial exclusion of black communities from community utility services in North Carolina, results can also inform research methods and provide insights for characterizing and understanding other underserved minority populations, such as colonias in South Texas and First Nations communities. This research provides an important contribution to the study of the access to safe and reliable water and sewer services for underserved and minority communities. The information gained from this project can be used to advance understanding of issues surrounding access to community water and sanitation for socially disadvantaged communities to inform various fields including microbiological testing of water sources, environmental management and policy, urban planning, and provision of public health services.

## Materials and methods

**Fig 1:**
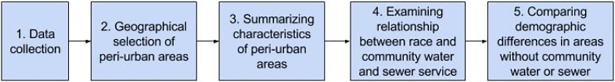
Main steps of the research methods.

Fig 1 shows the five main steps of this research project. First, water and sewer service data, demographic data, and geographic boundaries throughout North Carolina were collected from existing statewide sources (Table 1). Second, data were geographically selected to represent areas outside of municipal boundaries but within ETJs in order to define peri-urban areas on the outskirts of North Carolina municipalities containing communities that may be underbounded and lacking community water and sewer services. Third, summary statistics were calculated for peri-urban areas and compared to municipal areas. Fourth, logistic regression was performed to evaluate the statewide relationship between race and community water and sewer services in peri-urban areas. Fifth, and finally, areas lacking both community water and sewer services were identified, and demographic differences in these areas were summarized. All statistical analyses were performed in *RStudio 1*.*0*.*153* (RStudio, Boston, MA, USA). All geographic data manipulation, mapping, and selection was performed in *ArcMap 10*.*1* (ESRI, Redlands, CA, USA), and all shapefiles were projected into a uniform projection (NAD 1983 StatePlane NC FIPS 3200 [Meters]).

**Table 1:** Data types used and corresponding sources.

Data Type	Level(s)	File Type	Data Source	Information Sought
1997 Water Distribution Pipes and Sewer Pipes	County	Shapefile	North Carolina Rural Economic Development Center, 1997 [26, 27]	Location of water and sewer pipes to determine overlap with census blocks
2000 Census Blocks	Block	Shapefile	Manson S, Schroeder J, Van Riper D, Ruggles S, 2017 [28]	Geographic location to determine overlap with water and sewer pipes and area to calculate population density
2000 Census Demographic Data:		CSV	Manson S, Schroeder J, Van Riper D, Ruggles S, 2017 [28]	Summary statistics and variables for regression models
Total Population	Block, Place, County, Tract			
Race	Block, Place, County			
Median Home Value	Block Group, County			
Median Household Income	Block Group, County			
Urban and Rural Status	Tract, County			
2000 County Boundaries	County	Shapefile	Manson S, Schroeder J, Van Riper D, Ruggles S, 2017 [28]	Area to calculate population density of counties
2015 Extraterritorial Jurisdiction Boundaries	Municipality	Shapefile	Paul Black, Asheville MPO* Coordinator	Blocks that are in Extraterritorial Jurisdictions
1994 Municipal Boundaries	Municipality	Shapefile	North Carolina Department of Transportation, 1994 [29]	Blocks that are in municipalities

* MPO = Metropolitan Planning Organization

In this study, community water sources are defined as piped water sources from private companies or public utilities, while on-site wells are considered individual water sources. Similarly, community sewer service refers to piped public sewer services, while private on-site septic tanks are considered individual sewer systems. The ETJ is used to define peri-urban areas adjacent to municipalities. The term municipality is used to refer to incorporated cities, towns, and villages in North Carolina that have elected officials. Urban and rural populations are defined in this study according to the Census Bureau’s definitions, in which “urban” populations reside in a census block with a population density of generally at least 500 people per square mile that falls within a group of census blocks consisting of at least 2,500 total people. “Rural” populations are defined as residing in any area that does not fit the definition of “urban” [30, 31].

### Data collection

The most complete, consistent state-wide data available on community water and sewer service access at sufficient spatial resolution is a collection of 1997 water and sewer distribution pipe shapefiles from the North Carolina Rural Economic Development Center (NCREDC) [26, 27]. The water and sewer pipe shapefiles were mapped by individual system owners and were originally intended to facilitate planning, siting, and impact analysis in North Carolina counties. Though the 1997 water and sewer distribution pipe database used is nearly 20 years old, it is the most recent data source that is available statewide, consistently reported across counties, and at a geographical scale appropriate for this analysis. Three other potential data sources were considered but found to be unsuitable for this project:

1. utility-reported service boundaries, which contained more up-to-date data, but which display broad, overly inclusive service areas (for example, delimiting an entire county as served by the utility, even when other data sources indicate areas within the county lacking utility service);
2. tax parcel records from counties indicating utility service status, which also contain more up-to-date data, but which are only available from a few counties; and
3. the 1990 US Census (the last census to include questions about water and sewer service), which only shows data at the census block group level, which is too spatially coarse to identify racial differences.

**Fig 2:**
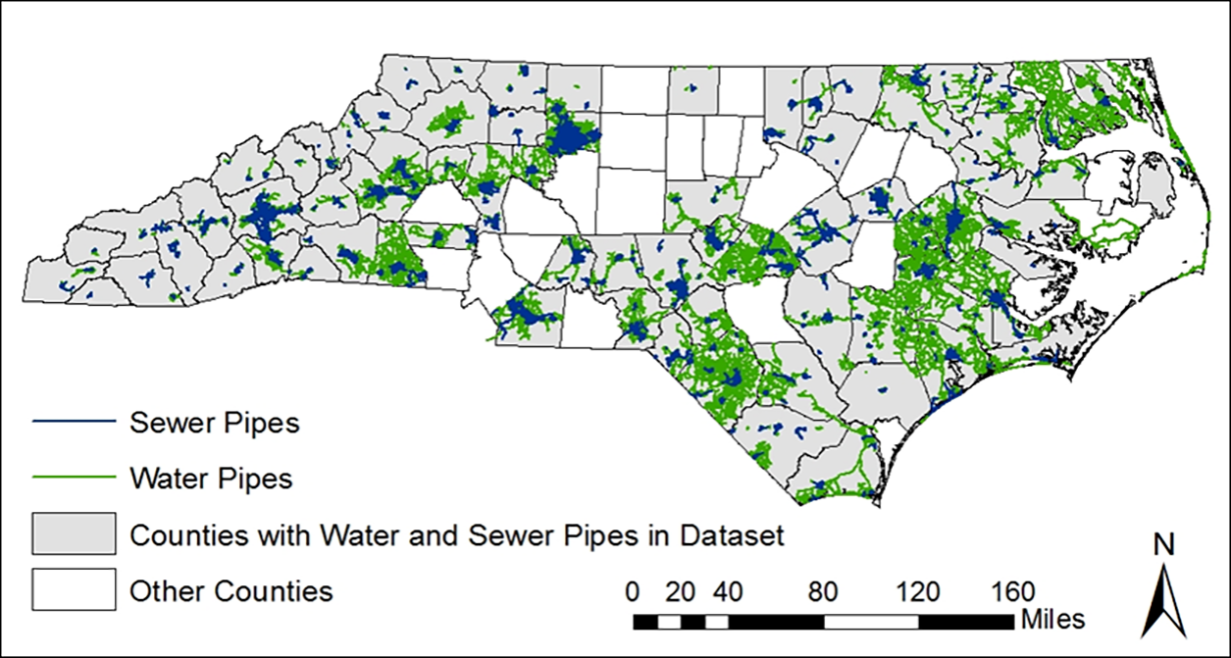
Water and sewer pipes in the 75 North Carolina counties included in the NCREDC dataset.

Therefore, the 1997 water and sewer distribution pipe shapefiles from the NCREDC were selected as the basis for this study as the most complete and reliable data source for a multi-county analysis. Fig 2 shows water and sewer pipes in the 75 of 100 North Carolina counties included in the NCREDC data set (see S1 File for counties included and not included in the dataset).

**Fig 3:**
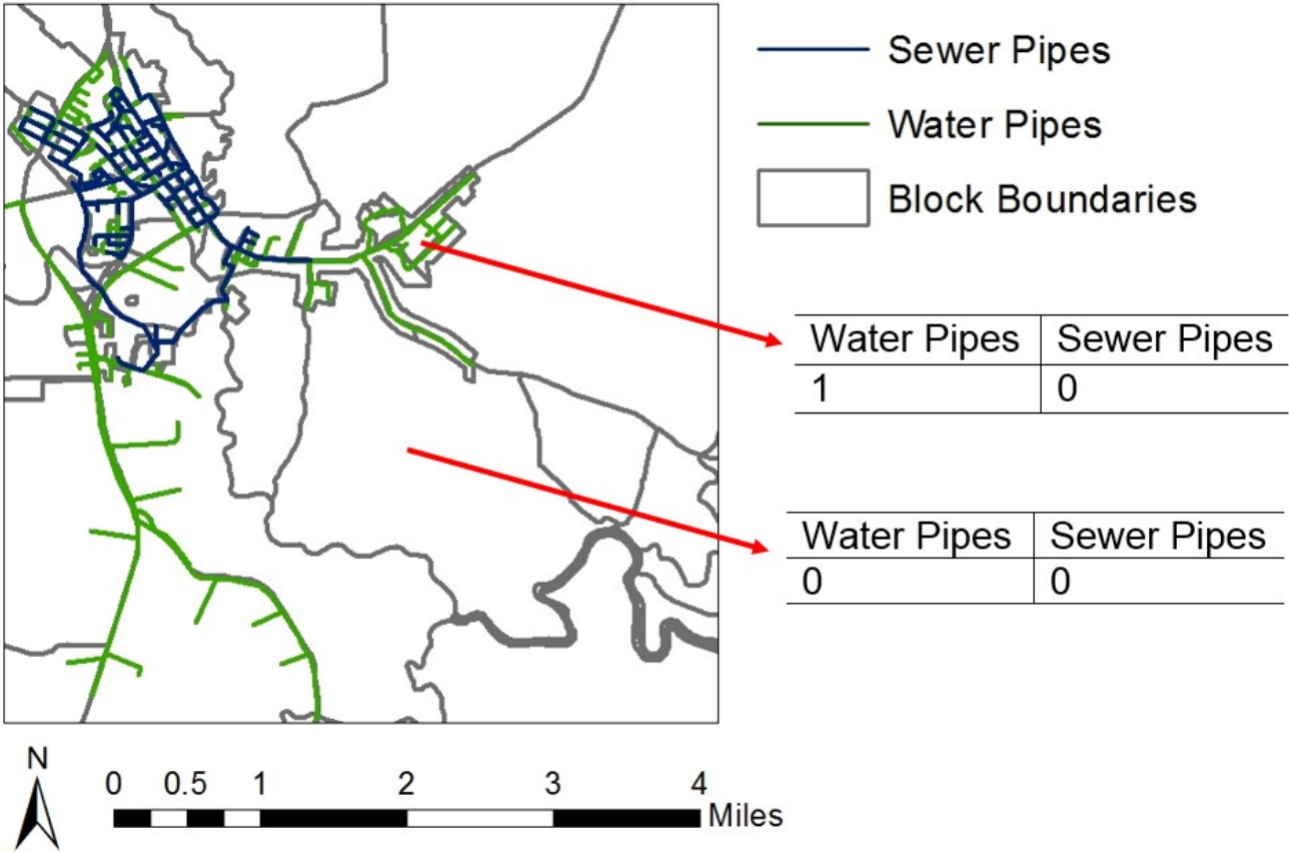
Assignment of binary variables reporting presence or absence of water and sewer pipes.

The NCREDC data were imported into *ArcMap*, and the “Select by Location” tool in *ArcMap* was used to determine if pipes intersect a census block. Community water and sewer access was then estimated at the census block level (using year 2000 census blocks) by creating two binary variables: one for water pipes and one for sewer pipes. These variables were assigned a value of one if the pipes intersect a census block and a value of zero otherwise. This variable assignment was performed in *ArcMap* by using the “Select by Location” tool to select all census blocks intersected by the water pipes and assigning a value of one for the water pipe variable for the selected census blocks. The “Switch Selection” tool was then used to select all census blocks not intersected by water pipes, and a value of 0 for the water pipe variable was assigned to these census blocks. The same process was used for sewer pipes to create a separate sewer pipe variable. Fig 3 depicts an example of the assignment of the binary water and sewer pipe variables.

Demographic data from the year 2000 at the census block, census block group, census tract, census place, and county levels were downloaded from the Minnesota Population Center, with racial categories as defined in the year 2000 United States Census [28]. ETJ boundaries were obtained from Paul Black, the Metropolitan Planning Organization Coordinator for Asheville, North Carolina. Municipal boundaries from the year 1994 were obtained from the North Carolina Department of Transportation [29].

### Geographical selection of peri-urban areas

The 1997 water and sewer pipe data were joined by location in *ArcMap* to the year 2000 census block boundaries, ETJ boundaries, municipal boundaries, and the corresponding demographic data described above.

**Fig 4:**
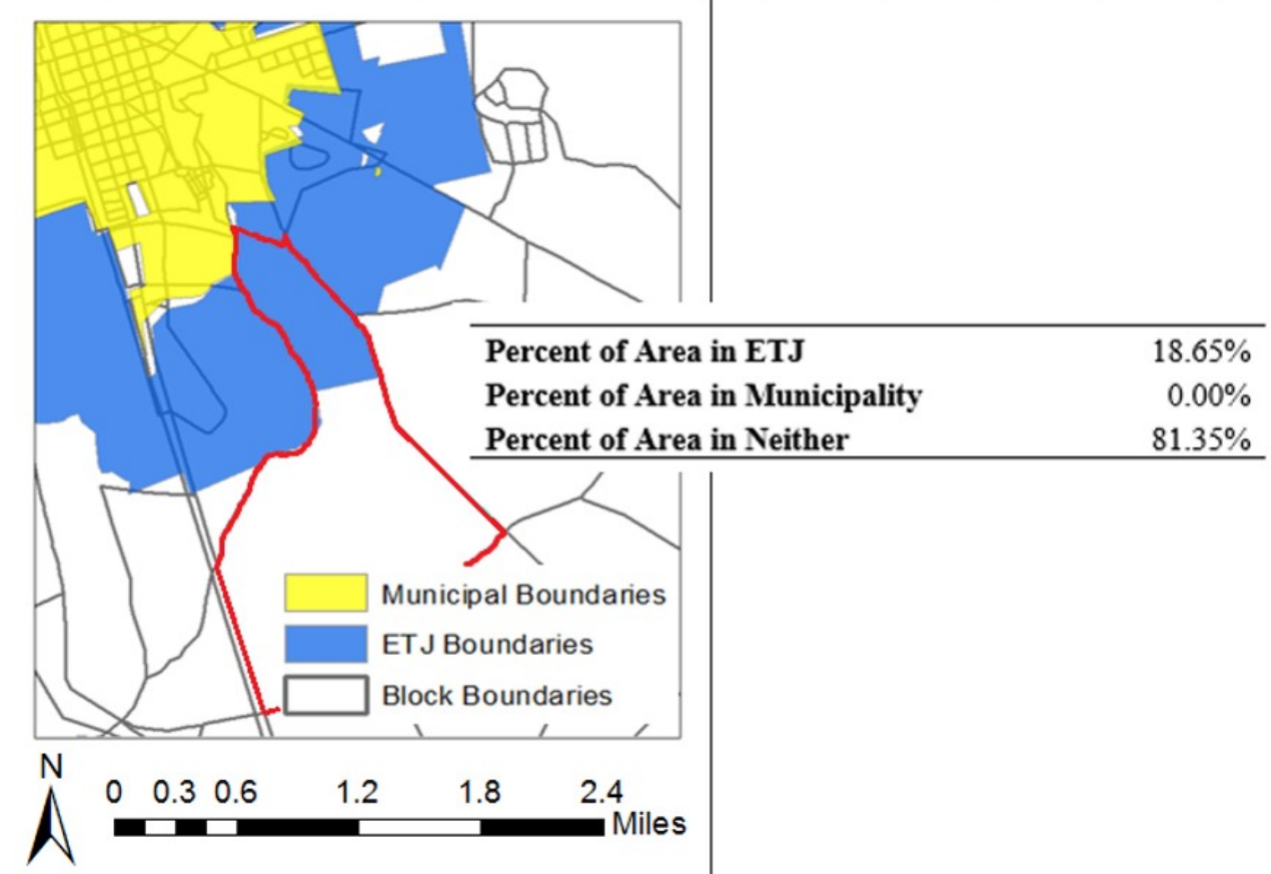
Calculated area in ETJ, municipality, and neither for a sample census block.

**Fig 5:**
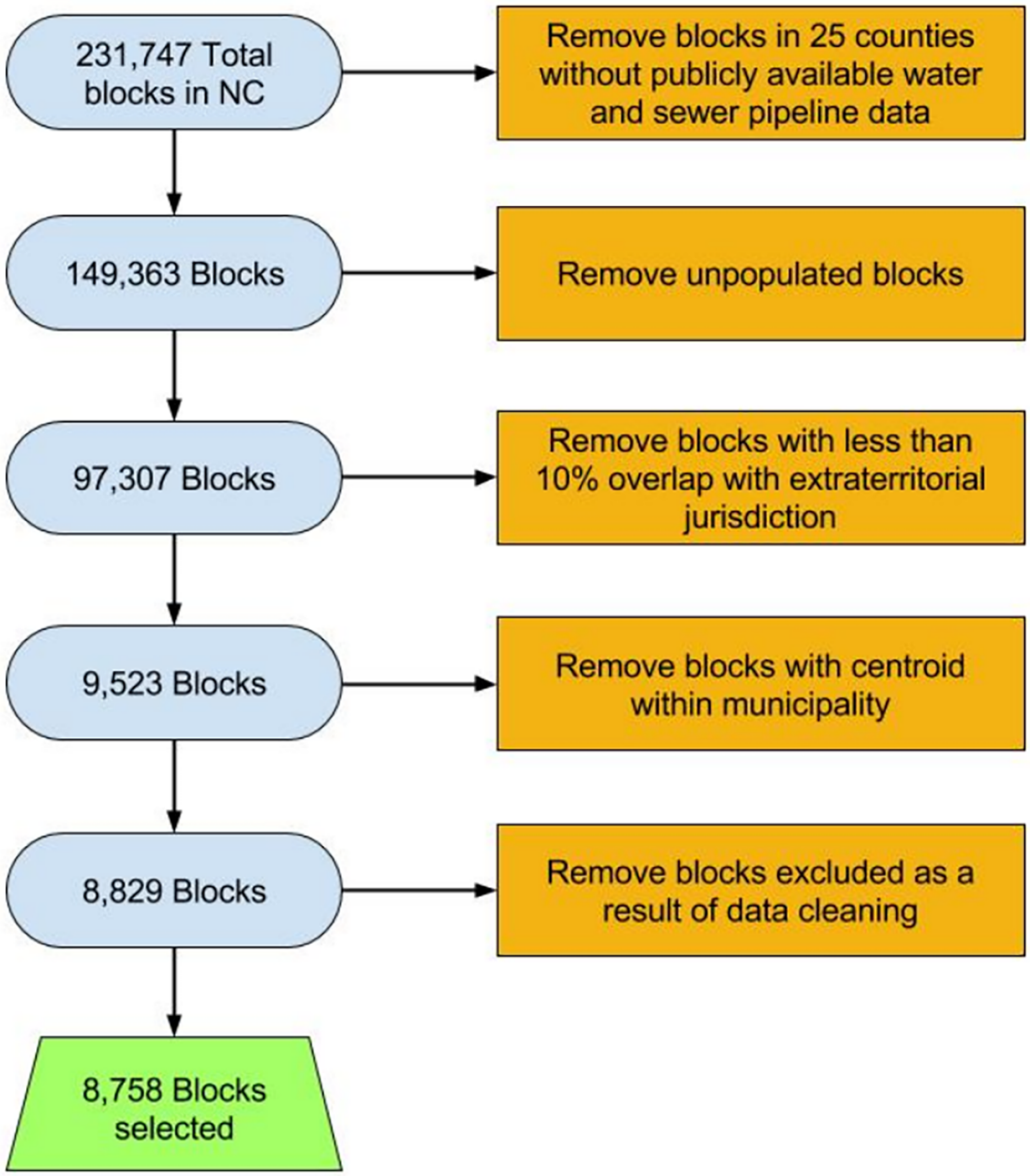
Selection criteria for North Carolina census blocks in peri-urban areas.

In order to select peri-urban census blocks in ETJs but outside of municipal boundaries, the percentages of land area for all year 2000 census blocks overlapping with the ETJ and with a municipality were calculated in *ArcMap* using polygon shape areas. The on-line supplementary information provides further detail on these calculations (S2 File). Fig 4 shows the calculated areas in the ETJ and municipality for a sample census block. The values shown in this figure refer to the census block outlined in red. The census block, municipal, and ETJ boundaries, as well as derived attributes, were then used to select a subset of all census blocks in North Carolina representative of peri-urban areas. The set of year 2000 census blocks for analysis was selected from an original data set containing all 231,747 blocks in North Carolina. The final 8,758 census blocks selected for analysis were chosen, as shown in Fig 5, by removing: (1) census blocks in counties not included in the NCREDC data set, (2) unpopulated census blocks, (3) census blocks with less than 10% overlap with an ETJ, (4) census blocks with centroids located within a municipality, and (5) census blocks excluded as a result of data cleaning. Census blocks with less than 10% overlap with an ETJ were excluded in order to obtain a sample of census blocks located in the ETJ rather than in rural areas or municipalities and based on visual inspection of differences in resulting block selections using different thresholds. Census blocks with centroids located within a municipality were excluded in order to ensure that the majority of each selected census block was in the ETJ and not in a municipality.

For a comparison, census blocks located in municipalities in the 75 counties included in the analysis were geographically selected as well. This dataset of 428,951 municipal census blocks was created by selecting census blocks with centroids located in a municipality, removing census blocks in the 25 counties not included in the analysis, and removing unpopulated census blocks.

Additionally, the percent white in the adjacent municipality for each census block in the selected peri-urban areas was calculated to use as a variable in the regression analysis assessing the relationship between race and community water and sewer service access. Percent white in the adjacent municipality was calculated by first performing a “Spatial Join” in *ArcMap* between the ETJ boundary file and the selected peri-urban census blocks to assign the municipality value from each ETJ to intersecting census blocks. Then, in *RStudio*, the peri-urban census block file with the new field for adjacent municipality was joined with 2000 census data containing racial composition data for the adjacent municipality (using data from census-defined places, which includes all incorporated communities).

### Summarizing characteristics of peri-urban areas selected for analysis

Data for the selected 8,758 peri-urban census blocks were imported into *RStudio* for analysis. The total number of individuals lacking community sewer service, lacking community water services, lacking both community water and sewer services, and lacking at least one of the two services was estimated by summing the populations of census blocks that did not have pipes intersecting them. Summary statistics for the peri-urban areas selected for analysis were also compared to comparable statistics for municipal census blocks.

### Regression analysis

The dependent variables used in the census block-level regression analyses were whether a water pipe, a sewer pipe, or at least one of the two types of pipes intersected the census block. For the dependent variable in the regression for at least one of the two types of pipes, a one was assigned to blocks intersected by at least one of the two types of pipes, and a zero was assigned to blocks lacking both pipes. The main independent variable of interest was the percentage of the census block population identified as black. Percent black was transformed into a categorical variable with the following racial composition categories, defined as follows:

1. “*100% Black*” (n = 459)
2. “*High % Black*” defined as 50% ≤ Percent Black < 100% (n = 849)
3. “*Medium % Black*” defined as 22% ≤ Percent Black < 50% (n = 753)
4. “*Low % Black*” defined as 0% < Percent Black < 22% (n = 1,720)
5. “*0% Black*” (n = 4,977 census blocks)

The category names above are used throughout the article to refer to these five racial composition categories. The five categories were chosen in order to provide cutoff values for race based on meaningful values and to assess for trends in access to community water and sewer services by race. Zero percent black had its own category because there were a large number (4,977) of peri-urban census blocks in this category. The 22% demarcation was selected because this is the mean black population proportion in North Carolina. The 50% cut-off was chosen to distinguish majority black census blocks, and 100% was selected to distinguish areas that are exclusively black (the mirror of 0% black).

In addition to race, additional independent variables were tested to control for socioeconomic status, population density, rural or urban status, race in the adjacent municipality, and region of the state. Socioeconomic variables tested were median household income and median home value. A regional designation (Piedmont, Coastal Plain, and Mountain), and interaction terms for race, income, and population density were also tested. The variables in the model with the lowest Akaike Information Criterion for predicting service access were chosen for all subsequent analyses. The final models were of the form

$$\ln\left({OR}_{i}\right)=\beta_{0}+\sum_{i=j}^{5}\beta_{j}B_{i}+\beta_{6}U_{i}+\beta_{7}D_{i}+\beta_{8}V_{i}+\beta_{9}W_{i}$$

where *OR*_*i*_ is the odds of a utility pipe intersecting census block *i*, *B*_*i*_ represent the five racial composition categories described above, *U*_*i*_ is the percentage of the population classified as urban in the census tract in which block *i* resides, *D*_*i*_ is the census block population density, *V*_*i*_ is the median home value in the census block group in which block *i* is located, *W*_*i*_ represents the percentage of the population in the adjacent municipality that is white, and the *ß*_*j*_ are regression parameters. Percent urban and median home value are at the census tract and census block group levels, respectively, because these variables are not reported at the census block level, and each census block was assigned the value of its corresponding census tract (for urban population) and block group (for median home value).

Standard errors and *p* values were calculated using robust variance estimators for all parameter estimates to account for clustering at the census block level. It is reasonable to expect that within this dataset, there may be a higher correlation of access to community water and sewer services for census blocks within the same geographical areas than for census blocks in different areas due to clustering; robust variance estimators adjust standard errors to account for this clustering effect. All regression analyses were performed in *RStudio* using the *glm* command and the binomial family. Robust standard errors were estimated using the *vcovHC* command in the “sandwich” package of *R* [32].

### Comparison of demographic differences in areas without community water or sewer

Median home value (used as an indicator of relative wealth and income) and population density were compared among census blocks in the different race categories lacking both community water and sewer service. This comparison of demographic characteristics in unserved census blocks focuses on census blocks lacking both community water and sewer services because these areas are at the greatest risk of negative health impacts due to the potential for cross contamination between private on-site septic tanks and wells.

## Results

### Characteristics of peri-urban areas selected for analysis

**Fig 6:**
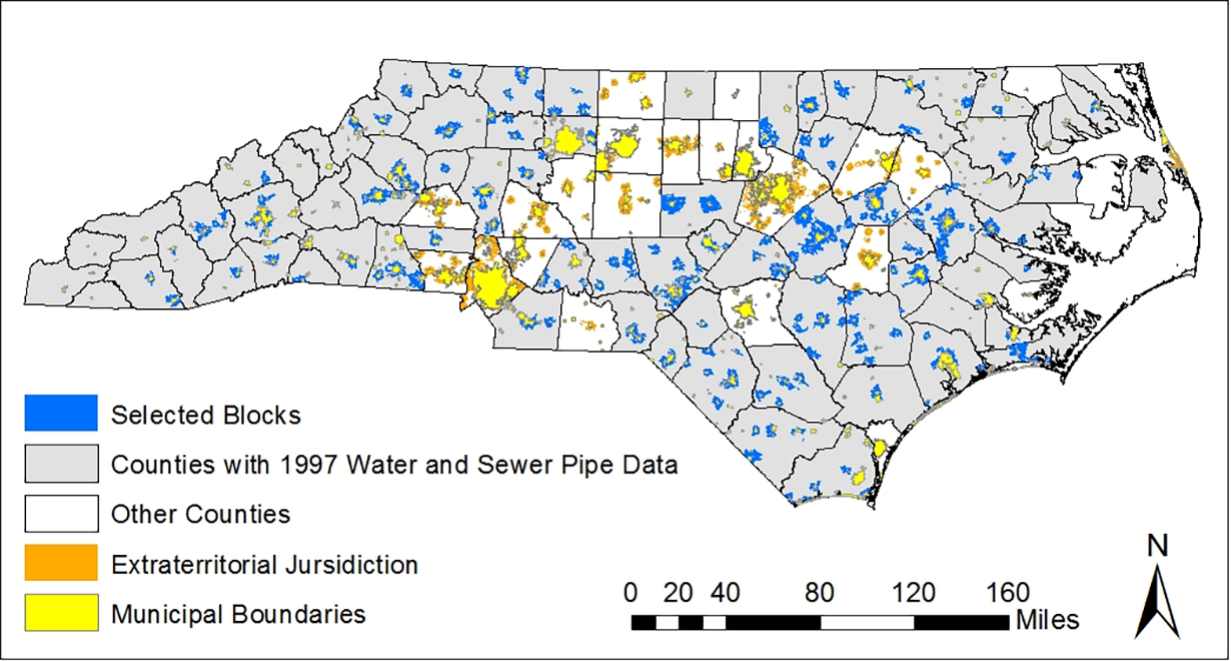
Census blocks in municipal extraterritorial jurisdictions in which access to water and sewer service was analyzed using the NC Rural Economic Development Center 1997 data set.

The peri-urban census blocks selected for this analysis are shown in Fig 6. Table 2 summarizes the characteristics of the 8,758 peri-urban census blocks selected for analysis, in comparison to populated municipal blocks in the 75 counties included in the dataset. Table 2 also summarizes at the county level the 75 counties included in the NCREDC dataset and the 25 counties excluded from the NCREDC dataset. The peri-urban blocks have a lower population density, percent urban, and percent black than the municipal blocks (all *p* < 0.001). The NCREDC, which focuses on economic strategies in North Carolina’s rural counties, created the water and sewer pipe shapefiles used in this analysis. As expected, the 75 counties included in the dataset have a lower population density and percent urban than the 25 counties excluded from the dataset (*p* < 0.01).

**Table 2:** Summary of census blocks included in analysis, municipal census blocks, counties included in the dataset, and counties excluded from the dataset.

	Peri-urban Blocks Selected (N = 8,758)	Municipal Blocks Selected (N = 32,693)	North Carolina Counties in Dataset (N = 75)	North Carolina Counties not in Dataset (N = 25)
Total Population	428,951	1,335,530	4,037,405	4,011,908
% Black ^*a*^	17%	25%	20%	25%
Median Household Income ^*a*^	$35,750 ^*b*^	$33,738 ^*b*^	$33,713	$38,355
Median Home Value ^*a*^	$87,204 ^*b*^	$89,149 ^*b*^	$92,285	$102,784
Population Density (people / mile^2^) ^*a*^	1,279	3,028	110	323
% Urban ^*a*^	47% ^*c*^	66% ^*c*^	29%	52%

*a—*calculated as a mean

*b–*Median household income and median home value used in block calculations are reported at the census block group level. Each block has been assigned the value of the block group it falls within.

*c–*Urban populations used in block calculations are reported at the census tract level. Each block has been assigned the value of the census tract it falls within.

This analysis finds that as of 1997, among peri-urban census blocks analyzed, an estimated 67% of the population were unserved by sewer pipes, 33% were unserved by water pipes, and 28% lacked both water and sewer pipes, as shown in Table 3. All told, as of 1997, 307,470 (72%) residents in these peri-urban areas lacked at least one of the two types of pipes (community water or sewer).

**Table 3:** Populations unserved by community water and sewer in selected peri-urban areas.

	Number of Blocks (%)	Total Population (%)
Lack Sewer Pipes	6,551 (75%)	286,965 (67%)
Lack Water Pipes	4,113 (47%)	140,931 (33%)
Lack both Water and Sewer Pipes	3,602 (41%)	120,426 (28%)
Lack at Least one Type of Pipe (Water or Sewer)	7,062 (81%)	307,470 (72%)
Total in Selected Peri-Urban Areas (75 Counties)	8,758	428,951

Note: the percentages in this table do not add up to 100% because the categories are not exclusive of one another.

### Relationship between race and community water service

Using logistic regression to explore the relationship between race and access to community water pipes and sewer pipes in peri-urban areas, we found statistically significant associations between the racial composition of census blocks and access to these infrastructure services.

Table 4 shows that areas in the “low % black” category have the highest odds of water service: odds of water service in these areas are 85% higher than in census blocks that are 100% black (*p*<0.001). Odds of water service in census blocks in the “medium % black” and “high % black” categories are also over 40% higher than in census blocks that are 100% black (*p*<0.01), but lower than in areas that are in the “low % black” category. Census blocks with no black residents have lower odds of water service than blocks that are 100% black, but this difference is not statistically significant (*p* = 0.928).

**Table 4:** Factors related to odds of having water pipes in selected peri-urban census blocks (N = 8,758).

	Odds Ratio	Regression Coefficient	*p* Value ^*r*^
Intercept	**1.85**	0.613	8.69 x 10^−7^
“100% Black” (reference level)	1.00	0.000	N/A
“High % Black” (50% ≤ Percent Black < 100%)	**1.42**	0.349	0.00325
“Medium % Black” (22% ≤ Percent Black < 50%)	**1.44**	0.363	0.00282
“Low % Black” (0% < Percent Black < 22%)	**1.85**	0.613	1.86 x 10^−8^
“0% Black”	0.991	-0.00922	0.928
Percent Urban ^*a*^	**2.18**	0.778	2.53 x 10^−31^
Population Density (100 people / mile^2^)	**1.00**	-7.05 x 10^−5^	1.04 x 10^−4^
Median Home Value ($10,000)^*b*^	**0.969**	-0.0314	5.58 x 10^−6^
Percent White in Adjacent Municipality	**0.380**	-0.968	3.09 x 10^−13^

**Bolded Odds Ratios** are statistically significant.

*r***–**Robust p values are reported.

*a–*Percent urban is reported at the census tract level. Each block has been assigned the value of the census tract it falls within.

*b—*Median home value is reported at the census block group level. Each block has been assigned the value of the block group it falls within.

Other variables with statistically significant influences on access to community water pipes in peri-urban census blocks are the proportion of the population in the block’s census tract classified as urban, population density, the median home value of the block’s census block group, and the percent of the population that is white in the peri-urban area’s adjacent municipality (all *p*<0.001). As expected, odds of access to water pipes increase substantially in areas with higher proportions of residents classified as urban. Contrary to expectation, odds of access to water pipes decrease slightly as population density increases when controlling for other variables in the regression, but the effect size is very small (the odds ratio for a change of 100 people/mi^2^ is 1.00). The odds of access to water service also decrease as the median home value increases, suggesting that some homes relying on private wells may be in areas of high socioeconomic status. Every 10% increase in percent white in the adjacent municipality decreases the odds of water service in a peri-urban block by about 9.2%, and as percent white in the adjacent municipality increases from 0% to 100% the odds of water service decrease by 62% (OR = 0.380, *p* < 0.001). This suggests that municipalities with a greater proportion of white residents are less likely to extend water pipes into peri-urban areas.

### Relationship between race and community sewer service

Similar to the results for water service, blocks with no black residents have the lowest odds of access to community sewer service (Table 5). Unlike for water service, blocks with no black residents have statistically significant lower odds of access to sewer pipes than 100% black blocks (*p*<0.001), and the odds of sewer service were not statistically different among the other race categories. The effects of percent urban, population density, and median home value are in the same direction for sewer service as for water service. As the percent white in the adjacent municipality increases, the odds of access to sewer pipes decrease, but not significantly, unlike for water pipes (*p* = 0.907).

**Table 5:** Factors related to odds of having sewer pipes in selected peri-urban census blocks (N = 8,758).

	Odds Ratio	Regression Coefficient	*p* Value ^*r*^
Intercept	**0.494**	-0.704	2.81 x 10^−7^
“100% Black” (reference level)	1.00	0.000	N/A
“High % Black” (50% ≤ Percent Black < 100%)	1.09	0.0898	0.480
“Medium % Black” (22% ≤ Percent Black < 50%)	0.949	-0.0526	0.688
“Low % Black” (0% < Percent Black < 22%)	1.05	0.0463	0.690
“0% Black”	**0.693**	-0.367	9.78 x 10^−4^
Percent Urban ^*a*^	**1.65**	0.503	6.98 x 10^−10^
Population Density (100 people / mile^2^)	**1.00**	-9.36 x 10^−5^	0.00873
Median Home Value ($10,000)^*b*^	**0.963**	-0.0381	4.34 x 10^−6^
Percent White in Adjacent Municipality	0.983	-0.0171	0.907

**Bolded Odds Ratios** are statistically significant.

*r***–**Robust p values are reported.

*a–*Percent urban is reported at the census tract level. Each block has been assigned the value of the census tract it falls within.

*b—*Median home value is reported at the census block group level. Each block has been assigned the value of the block group it falls within.

### Relationship between race and access to any community water or sewer service

Because areas lacking both community water and sewer service are potentially more at risk of exposure to drinking water contamination than areas with access to at least one of the two services (due to the potential for cross contamination between private on-site septic tanks and wells), an additional logistic regression was conducted to assess the relationship between race and the odds of access to at least one of these services.

The results for this regression were similar to those for community water service. Census blocks in the “low % black” category have the highest odds of having access to at least one of the two types of service: odds of having at least one of these services in these areas are 74% higher (*p*<0.001) than in census blocks that are 100% black (Table 6). Odds of access to any community water or service were also over 25% higher in census blocks in the “medium % black” and “high % black” categories than in blocks that are 100% black (*p*<0.05). As for water pipes, the lowest odds of access to at least one of the services are in blocks with no black residents, but the difference between 100% black and 0% black did not reach statistical significance (*p* = 0.528). The effects of percent urban, population density, median home value, and percent white in the adjacent municipality are all in the same direction for access to any water or sewer service as for water service (all *p*<0.001).

**Table 6:** Factors related to odds of having either water or sewer pipes in selected peri-urban census blocks (N = 8,758).

	Odds Ratio	Regression Coefficient	*p* Value ^*r*^
Intercept	**2.55**	0.934	3.09 x 10^−13^
“100% Black” (reference level)	1.00	0.000	N/A
“High % Black” (50% ≤ Percent Black < 100%)	**1.37**	0.312	0.0104
“Medium % Black” (22% ≤ Percent Black < 50%)	**1.28**	0.250	0.0440
“Low % Black” (0% < Percent Black < 22%)	**1.74**	0.551	7.49 x 10^−7^
“0% Black”	0.937	-0.0654	0.528
Percent Urban ^*a*^	**2.18**	0.780	5.10 x 10^−30^
Population Density (100 people / mile^2^)	**1.00**	-6.54 x 10^−5^	9.92 x 10^−4^
Median Home Value ($10,000)^*b*^	**0.958**	-0.0424	1.28 x 10^−9^
Percent White in Adjacent Municipality	**0.423**	-0.860	2.27 x 10^−10^

**Bolded Odds Ratios** are statistically significant.

*r***–**Robust p values are reported.

*a–*Percent urban is reported at the census tract level. Each block has been assigned the value of the census tract it falls within.

*b—*Median home value is reported at the census block group level. Each block has been assigned the value of the block group it falls within.

### Home value by race in areas without any water or sanitation service

**Fig 7:**
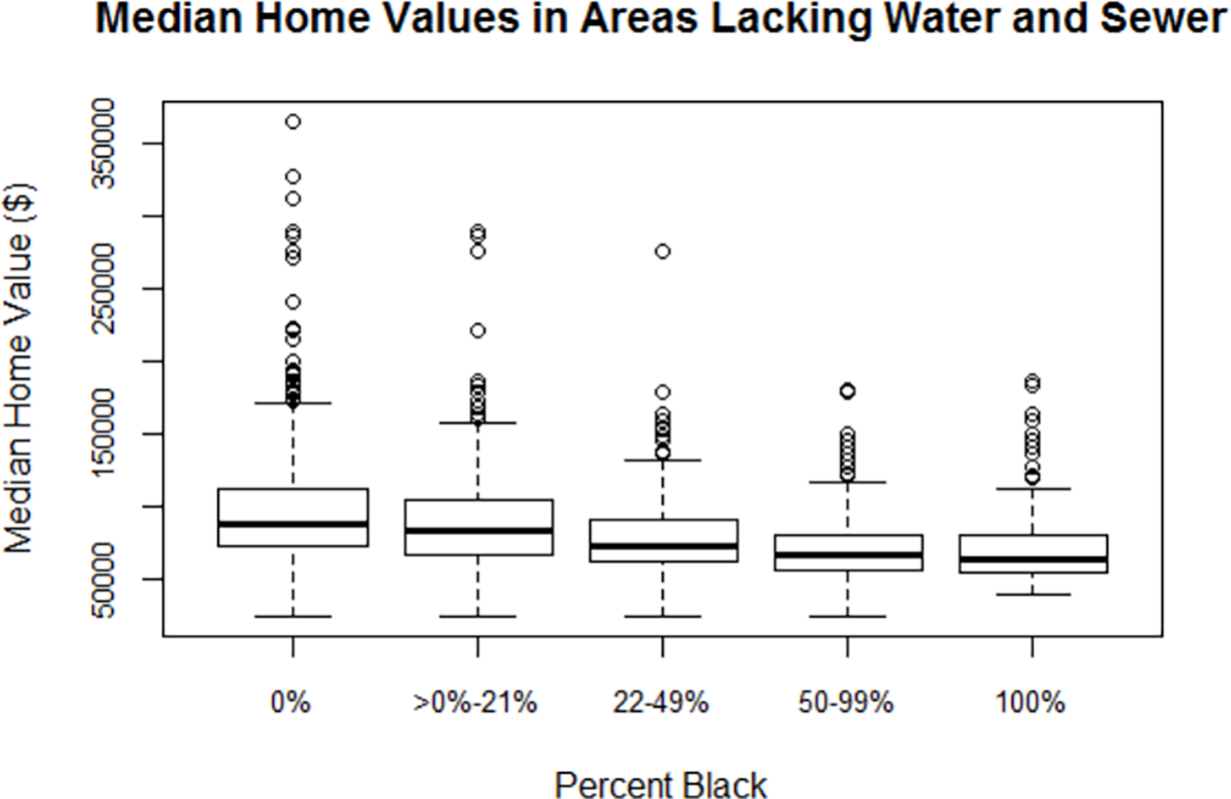
Median home values by race in peri-urban areas lacking community water and community water service.

To further explore the interesting result that access to community water and sewer service is lowest in census blocks that are either 100% black or 0% black, we compared median home values by race in areas lacking both community water and community sewer service. As Fig 7 shows, median home values in 100% black blocks lacking both community water and sewer service are lower than in 0% black blocks lacking both services: $71,500 as compared to $97,500 (*p*<0.001). Median home values decline as the percent of the population that is black increases. We also compared population density by race in areas lacking both community and community sewer service, but the differences in population density between the different race categories were not significant. The comparison of median home value and population density in the race categories showed similar trends in areas lacking just community water or just community sewer as well. These results suggest that compared to 0% black census blocks without water and sewer pipes, predominantly black census blocks are more economically challenged and therefore may have more difficulty affording maintenance of their private wells and septic tanks.

## Discussion

Motivated by a request from the former North Carolina State Public Health Director, we identified peri-urban areas across the state lacking community water and sewer service and analyzed the relationship between race and access to these services. We found that in areas other than those with no black residents, odds of having community water service or at least one of the two services were highest for census blocks with a small proportion of black residents and lowest in 100% black census blocks. Peri-urban census blocks that were 0% black had lower odds of having pipes than areas that were 100% black; however, this difference was only statistically significant for access to sewer pipes, not for access to water pipes or access to at least one of the two types of pipes. These 0% black census blocks lacking service were also wealthier than 100% black census blocks and blocks with mixed racial composition, as measured by median home value.

Overall, these results suggest two main trends: that there may be more racial disparities in access to community water than community sewer service in municipal ETJs and that there are two separate unserved groups of differing racial and socioeconomic status in peri-urban areas. These two unserved groups are: (1) lower-income black populations that may have been systematically excluded from municipal services on the basis of race during the era of legal racial segregation and (2) higher-income non-black populations. These findings support previous research revealing that some unincorporated areas are older communities suffering from poverty while others are new and suburban [11, 12]. The latter group may represent wealthier suburban areas that may be better able to afford to properly maintain private wells and septic systems in order to avoid waterborne diseases.

We also found that majority white municipalities are less likely to extend water and sewer pipes into peri-urban areas than are areas that are more racially mixed: as the percent of the population that is white in the adjacent municipality increases, the odds of access to these services in neighboring ETJs decrease. This result is consistent with a previous study of racial exclusion from municipal annexation, which found that primarily white municipalities were less likely to annex surrounding black communities [15]. Our finding supports the conclusion that the racial composition of a municipality may affect decisions regarding annexation and extension of services in peri-urban areas of North Carolina.

Our finding that peri-urban communities that were 100% black had lower odds of water service and lower odds of having at least one service (water or sewer) than areas with lower but nonzero black populations proportions is consistent with case studies documenting underbounding of black communities on the outskirts of specific cities, including Mebane, North Carolina, and Pinehurst, North Carolina, which were historically denied water and sewer services despite their close proximity to these services [3, 4, 6]. However, we did not find significant differences in access to sewer service alone among 100% black communities and those with lower but nonzero black population proportions. Like our multi-county analysis, a previous study of community water service in ETJs of Wake County, North Carolina, found that census blocks with 0% black population proportions had lower access to water service than census blocks with 1% to about 20% black population proportions [5]. However, the Wake County analysis found that census blocks with the highest black population proportions had the lowest access to water service, whereas our statewide study found that 0% black communities had slightly but not significantly lower access to service than communities that were 100% black. This difference may be attributed in part to differences in data used to support the analysis, or to differences in access to service or demographic characteristics of Wake County versus the state as a whole. The Wake County analysis was based on tax parcel data indicating the presence or absence of water service for each residential property, whereas our statewide analysis had to infer water service availability from the locations of water distribution pipes. The Mebane case study revealed that in that some communities, sewer pipelines traversed black census blocks without delivering service to those census blocks, so we may have over-estimated water and sewer service availability in some predominantly black census blocks [3].

### Limitations

The most important limitation of this analysis is that the most recent and complete statewide data source on water and sewer access in North Carolina in the post-Civil Rights era dates from 1997 and covers only 75 of North Carolina’s 100 counties. Because the 75 counties with data available on water and sewer pipes are primarily rural counties, and the 25 counties without this data available contain some of the larger cities in North Carolina, results may differ in some more urban counties. However, a Wake County (one of the 25 counties not included in this study) analysis of community water service in ETJs also found that as the percent black in census blocks increased, odds of community water service decreased [5]. Because the other data sources located with information on community water and sewer service access were not suitable for this analysis, it was not possible to perform a check for error in the water and sewer pipe data used. An additional limitation is that the ETJ boundaries (from 2015) and municipal boundaries (from 1994) are not from the same year as the census block boundaries (from 2000), so results are based upon the assumption that these boundaries did not undergo significant changes in the intervening years. The ETJ shapefile obtained is not for official use, and there may be errors of omission or inclusion in this dataset. Additionally, the binary coding of water and sewer pipes as a proxy for community water and sewer service relies on the assumption that individuals living in a census block intersected by pipes are served by these pipes. The “Select by Location” tool in *ArcMap* does not allow for sensitivity analysis, so this research also assumes that this tool accurately determined which census blocks are intersected by pipes. Because the selection criteria for peri-urban census blocks exclude from the dataset blocks with their centroid located within a municipality, but do not exclude blocks with a smaller amount of overlap with a municipality, some selected peri-urban areas may include a small proportion of census blocks representing partially municipal populations. Additionally, choosing a percentage overlap with the extraterritorial jurisdiction other than 10% as the cut-off for selection of peri-urban census blocks may have yielded different results, and a sensitivity analysis on this cut-off has not been performed. Because percent urban and median home value are not reported at the census block level, but rather at the census tract and census block group levels respectively, each census block was assigned the value of its corresponding census tract or block group. All calculations are based on assumptions of uniform population distribution across census blocks.

Despite these limitations, this analysis provides an important contribution to the study of underbounding and disparities in public health infrastructure service in North Carolina. This analysis identifies areas deserving closer inspection for potential municipal underbounding and for potential water and sewer service extension, informs priorities for future data collection, and provides a valuable quantitative report on the populations affected by lack of community water and sewer services.

### Generalizability

Results of this analysis are representative of peri-urban census blocks in rural counties of North Carolina, where peri-urban areas are defined as those outside of municipal borders but overlapping with the ETJ. The results of this research may be used as a basis and comparison for research performed in other areas of the southern United States and other areas with minority populations in peri-urban areas lacking community water and sewer services. The methods and data collection processes used can inform future state-wide studies of community water and sewer service in North Carolina as well as similar studies in other areas with social disparities in access to safe, regulated water and sewer services.

## Conclusions

New statewide data collection procedures are needed to prepare updated estimates of access to water and sewer service that cover all of North Carolina and that reflect current conditions. Ideally, an updated estimate would be based on current, household-level data. One possible way to generate such data would be to require that all counties include utility access information in their tax records. The North Carolina Department of Environmental Quality could use the resulting data to identify where water and sewer service extensions could be prioritized to serve populations that desire and would benefit from extension, perhaps through increased availability of grant and loan funds.

This study is the first systematic, multi-county analysis of access to community water and sewer services and the relationship between race and community water and sewer service in the southeastern United States. The results can be used to inform decisions regarding extension of community water and sewer services to under-served peri-urban areas. The findings of this research demonstrate the importance of accounting for two different groups living in peri-urban areas without community water and sewer services: lower socioeconomic status, mainly black neighborhoods and higher-income suburban neighborhoods with no black residents. In addition, this discovery suggests that the complexities and variations in unserved populations should be considered in other areas with minority populations unserved by community water and sewer services. Additionally, prioritizing extension of water first in peri-urban communities desiring services should be considered in North Carolina due to the lower cost of extending water than sewer service and the apparent greater disparities in access to community water than sewer service.

## Supporting information

S1 File List of counties included and excluded from water and sewer pipe files. (DOCX)

S2 File Description of calculation of percentages of land area of each block overlapping with the ETJ and the municipality. (DOCX)

S3 File Primary data used for census blocks in analysis. (CSV)

S4 File Image copyright and permissions statement. (DOCX)
