# Supplementary material for: Relationship between race and community water and sewer service in North Carolina, USA
Source: PLoS One. 2018 Mar 21;13(3):e0193225. doi: 10.1371/journal.pone.0193225 (PMC5862451; doi:10.1371/journal.pone.0193225)
Supplement: S1 File — (DOCX) [file pone.0193225.s001.docx]

Manuscript Title: **Relationship between Race and Community Water and Sewer Service in North Carolina, USA**

S1. List of counties included and excluded from water and sewer pipe files.

**Table S1**. List of the North Carolina counties included and excluded from the 1997 NCREDC water and sewer pipe files

| **75 Counties included in 1997 NCREDC files** | | | **25 Counties excluded from 1997 NCREDC files** |
| --- | --- | --- | --- |
| Alexander | Franklin | Onslow | Alamance |
| Alleghany | Graham | Pasquotank | Anson |
| Ashe | Granville | Pender | Cabarrus |
| Avery | Greene | Perquimans | Catawba |
| Beaufort | Halifax | Pitt | Cumberland |
| Bertie | Harnett | Polk | Currituck |
| Bladen | Haywood | Richmond | Davidson |
| Brunswick | Henderson | Robeson | Durham |
| Buncombe | Hertford | Rutherford | Edgecombe |
| Burke | Hoke | Sampson | Gaston |
| Caldwell | Iredell | Scotland | Gates |
| Camden | Jackson | Stanly | Guilford |
| Carteret | Johnston | Stokes | Hyde |
| Caswell | Jones | Surry | Mecklenburg |
| Chatham | Lee | Swain | Nash |
| Cherokee | Lenoir | Transylvania | New Hanover |
| Chowan | Lincoln | Union | Orange |
| Clay | Macon | Vance | Pamlico |
| Cleveland | Madison | Warren | Person |
| Columbus | Martin | Washington | Randolph |
| Craven | Mcdowell | Watauga | Rockingham |
| Dare | Mitchell | Wilkes | Rowan |
| Davie | Montgomery | Wilson | Tyrrell |
| Duplin | Moore | Yadkin | Wake |
| Forsyth | Northampton | Yancey | Wayne |
