# Supplementary material for: Relationship between race and community water and sewer service in North Carolina, USA
Source: PLoS One. 2018 Mar 21;13(3):e0193225. doi: 10.1371/journal.pone.0193225 (PMC5862451; doi:10.1371/journal.pone.0193225)
Supplement: S2 File — (DOCX) [file pone.0193225.s002.docx]

Manuscript Title: **Relationship between Race and Community Water and Sewer Service in North Carolina, USA**

S2. Description of calculation of percentages of land area of each block overlapping with the ETJ and the municipality.

Using *ArcMap,* new fields with a unique numerical ID for each municipality and ETJ were created in the municipality and ETJ shapefiles. Then the polygon shape areas for each municipality, ETJ, and block shapefile were calculated in new fields using the “Calculate Geometry tool”. A spatial union was created using the block, municipality, and ETJ shapefiles. The polygons in the resulting shapefile retained fields with the original calculated area of the input block polygon, as well as a field indicating the area of overlap between the block and the ETJ and municipality.

From the constructed shapefile, the “Summary Statistics” tool was used with the sum of shape area as the “field.” This summary statistic file was then imported into Microsoft Excel, where a pivot table was created with GISJOIN codes as “Rows,” area as “Values” and the ETJ and municipality IDs as “Columns.” This table was then re-joined to the attribute tables in *ArcMap*. The percent of area for each block in the ETJ, municipality, or neither was calculated by dividing the areas of overlap with the ETJ and municipality by the original block area.
