# Supplementary material for: Relationship between race and community water and sewer service in North Carolina, USA
Source: PLoS One. 2018 Mar 21;13(3):e0193225. doi: 10.1371/journal.pone.0193225 (PMC5862451; doi:10.1371/journal.pone.0193225)
Supplement: S4 File — (DOCX) [file pone.0193225.s004.docx]

The author, Hannah Gordon Leker, created all figures in the manuscript entitled “Relationship between Race and Community Water and Sewer Service in North Carolina, USA” and is the original copyright owner of these figures. PLOS ONE has permission to publish these figures under the Creative Commons Attribution License (CCAL), CC BY 4.0.
